# Premutation Females with preFXTAS

**DOI:** 10.3390/ijms26062825

**Published:** 2025-03-20

**Authors:** Valentina Liani, Carme Torrents, Elisa Rolleri, Nor Azyati Yusoff, Narueporn Likhitweerawong, Sydney Moore, Flora Tassone, Andrea Schneider, Ellery Santos, Hazel M. B. Biag, James A. Bourgeois, Kathryn E. Unruh, Matthew W. Mosconi, Randi J. Hagerman

**Affiliations:** 1 Medical Investigation of Neurodevelopmental Disorders (MIND) Institute, University of California Davis Health, Sacramento, CA 95817, USA; valentina.liani@aopd.veneto.it (V.L.); carme.torrents.fenoy@gmail.com (C.T.); elisarolleri@gmail.com (E.R.); nyusoff@ucdavis.edu (N.A.Y.); nlikhitweerawong@ucdavis.edu (N.L.); sydmoore@ucdavis.edu (S.M.); ftassone@ucdavis.edu (F.T.); anschneider@ucdavis.edu (A.S.); ersantos@ucdavis.edu (E.S.); hbbiag@ucdavis.edu (H.M.B.B.); 2 Department of Women’s and Children’s Health, University of Padova, 35122 Padova, Italy; 3 Unit of Pediatric Neurology, Department of Pediatrics, Hospital Universitari Parc Taulí, 08208 Sabadell, Spain; 4 Pediatric Neuropsychiatry Unit, Catholic University, 00153 Rome, Italy; 5 Department of Pediatrics, Faculty of Medicine and Health Sciences, Universiti Putra Malaysia, Seri Kembangan, Serdang 43400, Selangor, Malaysia; 6 Department of Pediatrics, Faculty of Medicine, Chiang Mai University, Chiang Mai 50200, Thailand; 7 Department of Biochemistry and Molecular Medicine, School of Medicine, University of California Davis Health, Sacramento, CA 95616, USA; 8 Department of Pediatrics, School of Medicine, University of California Davis Health, Sacramento, CA 95616, USA; 9 Department of Psychiatry and Behavioral Sciences, University of California Davis Health, Sacramento, CA 95817, USA; jbourgeois@ucdavis.edu; 10 Life Span Institute, Kansas Center for Autism Research and Training, and Clinical Child Psychology Program University of Kansas, Lawrence, KS 66045, USA; katunruh@ku.edu (K.E.U.); mosconi@ku.edu (M.W.M.)

**Keywords:** premutation, FXTAS, *FMR1* gene, neurological problems, neuropsychiatric problems

## Abstract

Fragile-X-associated tremor/ataxia syndrome (FXTAS) is a progressive neurodegenerative disorder associated with the *FMR1* gene premutation, characterized by the presence of 55 to 200 CGG triplet repeat expansions. Although the initial symptoms of FXTAS typically manifest in males around the age of 60 with motor symptoms and cognitive deficits, the presentation and progression in females differ. Women, in fact, exhibit a higher prevalence of neuropsychiatric symptoms, with an earlier onset compared to the motor symptoms observed in men. The following article reports on ten cases of women with a diagnosis of *FMR1* gene premutation, originating from two medical centers. All the women in the study exhibited neuropsychiatric symptoms and subtle neurological signs as common features. Symptoms typically observed in the male population, such as tremors and cerebellar ataxia, were either absent or significantly reduced in the female cohort. Conversely, there was a higher prevalence of neuropsychiatric symptoms among the women. Neurocognitive impairment was only minimally evident, with mild executive dysfunction and memory complaints noted in a subset of cases. For this reason, we propose the terminology *preFXTAS* or prodromic *FXTAS* to define a clinical presentation in women characterized by early manifestations of FXTAS that do not entirely fulfill the established diagnostic criteria but exhibit MRI evidence of white matter alterations suggesting the initiation of the disease process. The study underscores the importance of establishing new diagnostic criteria for FXTAS and, at the same time, developing new biomarkers and interview checklists/assessment scales dedicated to females.

## 1. Introduction

Individuals with the premutation of *FMR1* have 55 to 200 CGG repeats in the 5′ region, and their blood level of *FMR1* mRNA is elevated compared to controls without the premutation [1]. The elevated mRNA leads to RNA toxicity, followed by oxidative stress, mitochondrial dysfunction, inflammation, and the early death of neurons and astrocytes, leading to the neurodegenerative disorder fragile-X-associated tremor/ataxia syndrome (FXTAS) [1,2]. Although the onset of FXTAS typically begins in the 60s [3], males are more severely affected than females, with both cognitive and motor deficits [1,4]. However, females with FXTAS have more severe psychiatric problems that progress faster than males with FXTAS [5]. The prevalence of FXTAS has been reported to be at 8 to 16% in aging female carriers [6], but in males, it is higher, occurring at about 40% overall, but the prevalence increases with age to about 75% in the 80s [1].

There is evidence that some effects of RNA toxicity in premutation carriers can begin in childhood with visual–spatial deficits [1,7], anxiety [1], and Attention Deficit Hyperactivity Disorder (ADHD) [8,9]. In some cases, autism spectrum disorder can occur in young males with the premutation [10], but this is far less common in females with the premutation [11]. Females are less affected by the premutation because the normal X chromosome buffers the toxicity and severity of symptoms. However, about 20% of female carriers experience fragile X-associated primary ovarian insufficiency (FXPOI), meaning menopause before the age of 40 [12]. Neuropsychiatric and physical studies have shown that neurological deficits, such as motor sway in balancing tests [13], increased grip force variability during precision gripping [14], instability in tandem walking [4,15,16], central sensitivity syndrome [15], spasmodic dysphonia [17,18], and memory/ executive function deficits [19,20,21], can occur before the onset of FXTAS in carriers. In addition, other premutation-associated conditions (FXPAC), including neuropsychiatric disorders (FXAND), such as depression, anxiety, insomnia, and autoimmune problems, are common in females [1,22].

FXTAS was first reported in females in 2004 [1], and white matter disease and eosinophilic intranuclear inclusions of FXTAS occur in females and males [1,23]. However, in a study of over 50 females with FXTAS, Schneider et al. (2020) found that the MRI diagnostic features of FXTAS in males, the middle cerebellar peduncle (MCP) sign, occurred in only 9% of females compared to 60% in males [24]. The diagnostic criteria of FXTAS were created by the symptoms seen in males but not in females [1]. These criteria were modified by Hall et al. (2014), who added the neuropathy symptoms and the splenium sign of white matter hyperintensities [25,26], which is the most frequent MRI finding in females [24]. It is unclear if the diagnostic criteria for females are actually the same as those published for males (see Table 1). These criteria are very dependent on the presence of tremors and/or ataxia, but the major Central Nervous System (CNS) criteria are important because they visually demonstrate direct toxicity to the brain, leading to white matter disease in characteristic places such as the MCP sign and the splenium sign, in addition to brain atrophy. The phenotypic variability in premutation alleles is related to the size of the CGG repeat and the Activation Ratio (AR), meaning the percentage of cells that have the normal allele as the active allele. There are also secondary effects from modifier genes and environmental effects [1,27]. For instance, only about 20% of premutation females have early menopause, and the highest risk category includes those with repeats between 75 and 95, and smoking can also influence the age of onset of FXPOI [1].

Here, we report several cases of aging female carriers with neurological and/or neuropsychiatric features that do not meet the diagnostic criteria of FXTAS; however, there is white matter disease in the splenium and, in some cases, elsewhere. Because they do not meet the diagnostic criteria of FXTAS developed in males, but their MRI shows that the disease process of FXTAS has started, we have termed their involvement as preFXTAS.

## 2. Materials and Methods

### 2.1. Subjects

Ten unrelated premutation females were evaluated from two different centers. Six of them were evaluated at the UC Davis Medical Center MIND Institute of Sacramento, Fragile X Treatment and Research Center, and four of them through the University of Kansas’ community partnerships and Fragile X clinic.

We collected a standardized medical history and physical examination by physicians. The medical history investigated past medical problems, family history, and developmental history, focusing on any neuropsychiatric disorder, social history, and medications used in the past and currently.

All the participants underwent *FMR1* DNA testing, a neurological exam, a brain MRI, and neurocognitive studies.

### 2.2. Molecular Measures: CGG Sizing, Activation Ratio, and FMR1 mRNA Expression

Molecular data were obtained from peripheral blood samples (3 mL), and the Genomic DNA (gDNA) was isolated from peripheral blood leukocytes using standard methods (Qiagen, Valencia, CA, USA). CGG repeat allele sizing of the *FMR1* gene was measured by Southern blot and PCR analysis, as previously described [29,30]. In the molecular report for FXS, two sets of numbers are typically provided:

(i) The first number is the repeat number in the normal allele: The normal alleles range from 6 to 44 repeats.
(ii) The second number is the premutation allele: This has a range between 55 and 200 repeats.

The fraction of the normal *FMR1* allele on the active X chromosome, also known as the Activation Ratio (AR), was assessed using both Southern Blot (Sb) and mPCR analysis. AR was evaluated as an indicator of the percentage of cells bearing the normal allele on the active X-chromosome via two separate Sb runs for all samples (technical replicates), as described in Tassone et al. [31]. It is a measure of the relative expression of the *FMR1* gene in cells. A ratio closer to 1 would indicate a higher level of normal alleles active, which implies a higher level of FMRP and lower level of mRNA, whereas a lower ratio suggests more premutation alleles active, which implies a higher level of mRNA and perhaps a lower level of FMRP. *FMR1* mRNA levels were measured using a qRT-PCR that used Assays-On-Demand (Applied Biosystems, Foster City, CA, USA), custom TaqMan primers, and probe assays, as reported in Tassone et al. [1].

### 2.3. Neuropsychological Testing

Cognitive and behavioral assessments included the Wechsler Assessment for Adults (WAIS IV), the Wechsler Abbreviated Scale of Intelligence (WASI), or the Stanford–Binet. The Montreal Cognitive Assessment (MoCA), the Mini Mental Status Examination (MMSE), and the Behavioral Dyscontrol Scale 2 (BDS2) were performed with most participants.

### 2.4. Magnetic Resonance Imaging (MRI)

A brain MRI was performed on all subjects. MRI pictures were evaluated by an expert medical professional (RJH) to study the presence of white matter hyperintensities (WMHs) on T2-weighted and FLAIR sequences in both cerebral and cerebellar (MCP, splenium sign) and the presence of brain atrophy, as they are major and minor neuroradiological criteria for FXTAS.

## 3. Case and Results

### 3.1. Case 1

Case 1 is a 71-year-old female premutation carrier. She has one son with fragile X syndrome (FXS), and her father died from FXTAS. She is a university professor. She has a history of insomnia as a child and, more recently, in adulthood, associated with sleep apnea; therefore, she uses CPAP.

Her menopause was in her 50s, and she has not been on hormonal replacement. She has had osteoarthritis in her fingers and in her feet. She has taken vitamin D in the past. She had tingling in her fingertips and in her toes, and she had a neuroma removed surgically in her right foot at 64 years old. She had carpal tunnel surgery at 67 years old. She has suffered from recurrent urinary tract infections (UTIs) in the past, and she uses nitrofurantoin when they still occur. Gastroesophageal reflux disease (GERD) has been treated with omeprazole. She has urgency for urination at times, and she has had some intermittent incontinence for stool as a result of an episiotomy. No autoimmune disease has been reported, but she sometimes experiences irritable bowel syndrome. She drinks alcohol 2–3 times/week, and she exercises regularly. She reports balance problems from 69 years old, but no falls have been reported. She described about 6 months ago an isolated slight tremor in her hand.

She suffered from major depression during the pandemic, and since then, she has been taking fluoxetine. She reported memory problems from 69 years old onward.

A cognitive assessment showed a superior IQ, with a full scale of 126 on the WAIS 4, an MOCA score of 25/30, a BDS2 of 20/27, and an MMSE of 30/30.

During the neurological exam, her tandem walking was unstable, but she took 10 steps, and no tremor was seen. Her exam was otherwise normal, although she has a positive snout reflex.

The MRI demonstrated normal middle cerebellar peduncles but white matter disease in the insula bilaterally and in the splenium of the corpus callosum, with one hyperintensity spot in the cerebrum and mild atrophy but normal ventricles. Her blood test inflammatory biomarkers (C-reactive protein and antinuclear antibodies (ANA) were negative.

### 3.2. Case 2

Case 2 is a 61-year-old female premutation carrier; her family history includes a son with a full mutation and a deceased father who had dementia but no other FXTAS symptoms. She has a history of anxiety and depression, was previously treated with fluoxetine, and currently manages with escitalopram and counseling. Academically, she holds two Master’s degrees.

She was diagnosed with FXPOI at age 35, for which she has been on hormone replacement therapy. At 51, she developed hypertension, treated with lisinopril, and she has sleep apnea managed with a CPAP machine. At age 59, she was diagnosed with narcolepsy, which is currently managed with mixed amphetamine salts (Adderall). She underwent two knee replacement surgeries and carpal tunnel surgery in the last three years. Following each anesthesia, her husband noted worsening cognitive issues, which had increased in the months previous to her last examination, particularly memory problems that worsened under stress. An MRI performed at that time revealed mild atrophy and slight white matter changes in the insula and splenium.

Upon examination, her blood pressure was elevated (158/96), and her BMI indicated obesity (41 BMI). Her neurological exam was mostly normal, except for absent DTRs in the knees (likely due to previous surgery) and slightly reduced ankle reflexes. A positive snout reflex lasting over four taps was noted, while the palmomental reflex was negative. There was no tremor during finger-to-nose testing, and her tandem gait was normal. Cognitive assessments showed an average IQ score and a MOCA score of 21/30, supporting her reports of memory issues. A psychiatric assessment confirmed significant generalized anxiety and a history of depression (see Table 2, Table 3 and Table 4).

### 3.3. Case 3

Case 3 is a 72-year-old female premutation carrier with 69 CGG repeats. She has two daughters, one of whom is also a premutation carrier. This daughter has a son diagnosed with fragile X syndrome. The patient’s mother had FXPOI, but neither of her parents exhibited signs of FXTAS.

The patient reported experiencing severe shyness and learning disabilities during childhood, particularly in mathematics. Her medical history is significant for a diagnosis of mixed connective tissue disorder (MCTD Table 3), including Sjogren’s syndrome, severe Raynaud’s phenomenon, and rheumatoid arthritis. She also suffers from severe osteoporosis, jaw degeneration, epiretinal disease, macular degeneration, and ocular migraines. Additionally, her psychiatric history includes major depressive disorder (currently in partial remission), anorexia nervosa at age 21, obsessive disorder, social phobia, specific phobia, and PTSD from past abuse.

At her last visit, the patient reported worsening balance problems that began intermittently every few months and have now progressed to a monthly occurrence. She has also experienced intermittent episodes of numbness in her toes over the past two years. Physical examination findings included a normal gait, including tandem walking, hypoactive reflexes in the upper extremities, and a positive snout reflex. Sensory tests, including vibration, position, cold sensation, and pinprick, were intact, and no tremors were observed.

**Figure 1:**
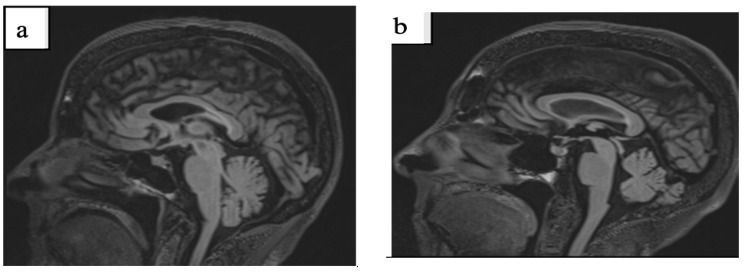

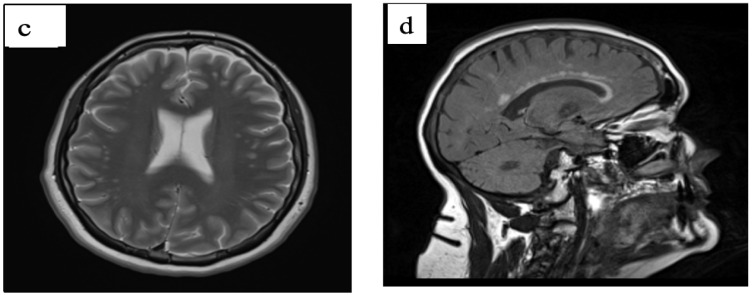
Splenium sign ((**a**): Case 3, (**b**): Case 5), white matter disease ((**c**): Case 6) white matter disease in the genu and splenium of the corpus callosum and in periventricular area ((**d**): Case 7).

Neurocognitive testing results revealed a Full-Scale IQ of 98, a MOCA score of 30/30, and a BDS-2 score of 23/27, indicating preserved cognitive function despite her neurological symptoms (Table 4). Brain MRI results showed mild cerebral atrophy with mild sub-insular, splenium, pons, and periventricular white matter hyperintensity (Figure 1a) (Table 5). Her blood test inflammatory biomarkers (C reactive protein and antinuclear antibodies) were negative.

The patient is currently taking calcium and magnesium supplements and sertraline for her depressive symptoms. She has also reported a moderate alcohol intake, consuming two glasses of red wine nightly for the past six months.

### 3.4. Case 4

Case 4 is a 52-year-old female premutation carrier with 114 CGG repeats. Her family history includes two children with fragile X syndrome and a father who had FXTAS, who died 5 years ago during spinal surgery. Academically, she holds a college degree. She reported anxiety problems, difficulty in falling asleep, and nighttime awakenings, associated sometimes with sweating or headaches, suggesting a possibility of sleep apnea; for that reason, we suggested a sleep study. She referred to depression; she does not take any medication and had 2 months of counseling in the past; we recommended that she start a selective serotonin reuptake inhibitor (SSRI). She complains of chronic fatigue, joint pain, and a migraine headache from about 38 years of age, associated with visual aura such as sparkling lights. She suffered from endometriosis; therefore, she underwent a partial hysterectomy at 42 years old. She never used hormonal replacement therapy, except estrogen gel, for 6 months. Osteoporosis has been denied, and she is taking calcium and vitamin D. She suffers from constipation (treated with fiber and laxatives) and frequent urinary infections. She has had rare urine incontinence in the last 2 years. She drinks alcohol during the weekend. She does not exercise, and no autoimmune disease has been reported; she complains of pain in her knees and hips for the last few years. She suffers from some dizziness when getting up fast, and she has had three falls in the last two years. She denies balance problems and attributed falls to feeling light-headed.

She has had numbness and tingling in her legs in the nighttime, and her feet have a burning sensation in bed three times a week in the last 2 years. She has noticed an intermittent tremor in both of her hands since age 50, which occurs about twice a week. She has reported memory problems in the last 3 years that are gradually worsening.

The neurological exam showed a very subtle subclinical intention tremor bilaterally during the finger-to-nose testing and a subclinical postural tremor, especially on the right side, with her arms extended, but no resting tremor. Gait was normal, and the tandem walking was fine for 10 steps, but she had a positive snout reflex with four taps. A Cognitive assessment showed an average IQ and a MOCA score of 22/30, and her BDS2 was 15/27, which can be compromised by her language comprehension difficulty. On the Structured Clinical Interview for DSM Disorders (SCID), she met the criteria for major depressive disorders, social phobia, and obsessive compulsive features, without meeting the Obsessive-Compulsive Disorder (OCD) criteria. Her MRI demonstrated several white matter hyperintensity spots in the frontal and parietal areas. Her splenium had some slight hyperintensities, but she did not have significant brain atrophy.

### 3.5. Case 5

Case 5 is a 75-year-old female premutation carrier with 79 CGG repeats. She has a 47 year old son with FXS with 196 CGG repeats. There is also a strong family history of fragile X mutations in her siblings, with two affected brothers and two brothers who had features of FXTAS.

The patient has had a history of migraines since age 16, with visual aura, but the migraines reduced once she reached her menopausal phase at the age of 43. The migraines, however, have become worse in intensity over the past 4 years. Treatment included rimegepant (Nurtec), triptans, and monthly Erenumab injections. She has undergone multiple surgeries, including tonsillectomy and adenoidectomy at 6 years old, gallbladder surgery, spinal disc surgery, CNS dural leak surgery, bilateral hip replacements, cataract surgery, and an L3-L4 spinal cleaning procedure, with complications including emesis and CNS dysfunction requiring additional hospitalizations. She also had a hysterectomy at 65 and a bladder lift.

Her current medications include statin, estrogen (dose unknown), Minoxidil, triptans, Erenumab, gabapentin, magnesium, CoQ10, B2, turmeric, and iron pills for restless leg syndrome (RLS). She also has chronic pain, arthritis, and frequent migraines. She reports memory decline similar to her peers, Raynaud’s syndrome, dry eyes (without Sjogren’s), and osteoporosis. She denies significant anxiety or depression. The patient experiences leg cramps at night, though magnesium has not helped. She denies tremors, though her daughter reports intermittent tremors and worsening handwriting for her. She has fallen three times in the past year while walking her dog, and her daughter noticed intentional tremors and memory decline, including difficulty recalling dates and her husband’s birthday.

She has been experiencing swallowing issues and hoarseness following a viral infection that caused unilateral vocal cord atrophy and underwent therapy with injections. She also reports decreased hearing, though it was deemed normal, and urinary incontinence, partially improved with pelvic PT therapy. Her energy levels have been low since her recent COVID-19 infection, and her exercise routine has decreased.

Her neurological exam shows some evidence of neuropathy, a subclinical tremor in her non-dominant hand, a very slight positional tremor bilaterally with her arms extended, and mild gait instability with tandem walking. Her brain MRI shows mild evidence of white matter hyperintensities in the splenium (Figure 1b), with a few additional spots in the cerebrum.

### 3.6. Case 6

Case 6 is a 60-year-old female premutation carrier with 114 CGG repeats who has two biological children, including one son with FXS and one daughter who is a premutation carrier. She also has five biological sisters, each of whom are premutation carriers. Her father was a known premutation carrier who passed away at age 94. He reportedly was diagnosed with Parkinson’s Disease and dementia. Case 6 reported that her father demonstrated increased emotional outbursts, balance issues, and tremors as he aged, so he likely had FXTAS. Her paternal grandmother showed memory problems and was age 99 years old when she passed away.

Case 6 has reported past and current discomfort when making eye contact and in social situations, and she also indicated that she believes she has shown socially inappropriate disinhibition throughout her life. She reported post-partum depression and anxiety in the past that lasted about 10 years. She also noted symptoms of attention-deficit/hyperactivity disorder (ADHD), though she never sought a clinical evaluation and, therefore, does not have a diagnosis of ADHD. She reported snoring during sleep and difficulties with waking up throughout the night that began at the age of 45 years. She has not been evaluated for sleep apnea. She had a hysterectomy at age 43 and believes she was menopausal prior to the procedure. She received estrogen and progesterone hormone replacement from ages 43 to 48. She has chronic sciatic nerve back pain with onset at age 55 years, as well as arthritis in her knees, hips, and hands. She endorsed balance issues that she believes have been present all her life, including regular tripping because of not lifting her feet sufficiently during walking. Currently, she experiences approximately two falls per year. She has swallowing and choking problems due to a growth in her throat that began at the age of 40 years. She endorsed memory problems that developed at age 40, including word-finding difficulties, and she experienced hearing loss beginning at age 55 years. She currently reports decreased stamina and muscle weakness in both her upper and lower extremities.

During a clinical exam, Case 6’s blood pressure was 135/71, and her heart rate was 84 bpm. No abnormalities were noted other than a slightly decreased reflex response and mild abnormalities when making smooth pursuit eye movements, drawing a spiral with her left hand, and standing on one foot during the FXTAS Rating Scale assessment (FXTAS-RS). No tremors or difficulties during tandem walking were noted during the exam. She reported mild difficulties getting off the floor when lying on her back, walking on uneven surfaces, walking on slippery surfaces, and walking for up to 20 min due to exhaustion. She reported moderate difficulty using stairs and major difficulty using stairs without a handrail. Her executive abilities appeared to be relatively intact during BDS-2 testing, though she showed mild difficulty in sequencing movements and when generating lists of alternating letters and numbers.

A T2-weighted MRI showed mild white matter disease in both the genu and splenium of the corpus callosum (Figure 1c), as well as mild cerebellar disease reflected by hyperintensities in cerebellar white matter and the cortex.

### 3.7. Case 7

Case 7 is a 70-year-old female with 69 CGG repeats and three biological daughters, all of whom are premutation carriers. Case 7 also has three biological grandsons diagnosed with FXS, as well as a grandson without FXS and a granddaughter who has not yet received genetic testing. She also has three biological sisters, two of whom are premutation carriers; the third sister has declined genetic testing but has a daughter who has an intellectual disability but no genetic testing. One of the sisters, who is a premutation carrier, has a daughter who is a carrier and two grandsons, including one who tested negative for the *FMR1* mutation. Case 7’s father died at age 59 of a cardiovascular incident as a complication of Type 1 diabetes, and his medical history is otherwise unknown. Her paternal great-uncle was diagnosed with Parkinson’s Disease, and she reports a suspicion of dementia for her paternal grandfather. Case 7’s mother is 87 and reportedly shows cognitive decline as well as difficulty balancing while walking; she has experienced multiple falls.

Case 7 identified concerns about recent declines in memory abilities (in the past 1–2 years). She also reported a history of violent outbursts (e.g., throwing items when upset), but she indicated that these episodes have been rare in recent years. She reports that, throughout her life, she has experienced anxiety during social interactions and when in new situations. She also recalled at least one prior panic attack prior to a public speaking event multiple years ago. She reports persistent loneliness beginning during the COVID-19 pandemic (~4 years ago). She received psychological counseling beginning at age 28 following the passing of her uncle, with whom she was close. She reports alcohol abuse from her late teens to her early 20s, though she has remained abstinent for the past 45 years. She also reported smoking 2–3 packs of cigarettes per day from about age 11 to 32 years, but she no longer smokes. Case 7 had a hysterectomy at age 39 but did not receive a diagnosis of FXPOI. She reports hypertension beginning at approximately age 62. Her blood pressure at the time of testing was 167/69. She also has an irregular heartbeat that was noticed in the last year during a study for sleep apnea. Case 7 is diagnosed with sleep apnea and now sleeps with a CPAP machine. She does not report any head or hand tremors, but she indicated that her handwriting has become less steady and that she has experienced more frequent tripping while walking during the past year. Case 7 reports having tested positive for COVID-19 at least five times, though all incidents were reportedly mild. She has developed difficulty smelling and tasting certain foods since her most recent COVID-19 infection.

During clinical examination, Case 7 did not show any prominent neuromotor signs, though she had mild difficulties during spiral tracing and made three deviations during tandem walking on the FXTAS-RS, but she otherwise was able to complete the ten required steps. She has also reported mild difficulties opening containers, getting off the floor when lying on her back, and using stairs without a handrail. Her executive abilities appeared to be unaffected during BDS-2 testing. She reported clinically elevated scores on the SCL-90 (T score > 70) hostility subscale, and there were mildly elevated (T score >60) on multiple subscales, including the obsessive-compulsive, interpersonal sensitivity, depression, phobic anxiety, and psychoticism subscales. Memory performance on standardized testing (California Verbal Learning Test, or CVLT-3) ranged from “borderline” (learning score) to “low average” (long-term memory and long-term recognition) to “average” (short-term memory).

Analysis of T2-weighted MRI data indicated multiple cerebral hyperintensities that were thought to possibly be the product of her sleep apnea and hypertension. White matter hyperintensities were seen in both the genu and splenium of the corpus callosum and periventricular areas (Figure 1d). A large CSF-filled lesion was observed in the posterior parietal cortex, extending to the corpus callosum, though the cause of this lesion or its functional significance could not be determined.

### 3.8. Case 8

Case 8 is a 71-year-old female with 102 CGG repeats in *FMR1*. She has reported current concerns related to sleep disturbance (difficulty sleeping through the night), osteoporosis and osteopenia, arthritis in her toes and shoulders, a history of early menopause (age 39 years), though this coincided with treatment for Hodgkin’s lymphoma and a history of being diagnosed with Raynaud’s syndrome and hypothyroidism.

During the clinical exam, Case 8 showed mild subclinical tremors during writing, as well as head tremors that she did not recognize. No other difficulties were noted during the FXTAS-RS, including during tandem walking. No memory or executive functioning issues were seen on standardized testing. No psychiatric concerns were reported. No other clinical concerns were noted.

In the MRI, mild white matter disease was seen in the pons, insular cortex, and, more broadly, in the cerebrum. White matter disease was also seen anterior to the lateral ventricles, though this disease was considered mild. Her ventricles were also moderately enlarged. Mild white matter disease was seen in the corpus callosum, especially in the genu and splenium.

### 3.9. Case 9

Case 9 is a 78-year-old female premutation carrier with 61 CGG repeats. She has one biological daughter who is a premutation carrier and one biological son who has not received genetic testing. Case 9’s daughter has three biological sons, one of whom has FXS, whereas the other two sons tested negative for the premutation/mutation. She also has one biological daughter who tested negative for the *FMR1* premutation. Case 9’s older biological sister died at age 75 and had a history of obesity, atrial fibrillation, and reported thyroid issues, though she never received genetic testing. Her younger sister (age 74) has been diagnosed with dementia, epilepsy, thyroid problems, sleep apnea, atrial fibrillation, and balance problems. The patient strongly believes her younger sister has FXTAS, but the sister disagrees, and her doctor has refused further testing. The sister has never completed genetic testing to determine their premutation status. Case 9’s younger brother (age 72) has been diagnosed with Alzheimer’s Disease; he does not have a history of any balance issues or tremors. His biological daughter is a premutation carrier. Case 9’s biological parents are both deceased; her mother died at age 63 of a congenital aneurysm, and her father died at age 70, reportedly due to an enlarged heart. Neither biological parent exhibited any problems with memory, balance, or walking. Case 9’s grandmother was diagnosed with Alzheimer’s Disease. No other family members have a known history of cognitive or motor issues.

Case 9’s medical background includes a long-standing history of obsessive-compulsive disorder (OCD), though she only recently received a clinical diagnosis (2 years prior to the present evaluation). She does not take any psychiatric medications, but she was in therapy for OCD for one year. She also reports a history of depression after her mother died in 1982, and she took an unknown antidepressant for six months around this time period. She has been on medication for hypertension since age 64, and her blood pressure during the evaluation was 140/88. She was diagnosed with atrial fibrillation in the previous year, but she has no current symptoms. She was diagnosed with breast cancer at age 42, for which she underwent chemotherapy. She reports back pain starting in the past year due to degeneration of the lower lumbar, and she has had arthritis in her hands and toes since age 64. She has had two surgeries within the last ten years (a hip replacement at age 68 and cataract surgery at age 72), but she reports no worsening of symptoms. She falls less than once a year, including a fall when tripping over wood in a garage at age 75 and a more recent fall while standing on a chair and reaching for an object. She reports hearing loss starting at age 73 and wears hearing aids.

During the clinical exam, Case 9 reported concerns with memory (forgetting names and words), but she indicated her desire to participate in research and was motivated by her interest in advancing knowledge of the *FMR1* premutation to benefit later generations (especially her daughter). She reported no tremor or balance concerns but showed multiple neuromotor issues during the FXTAS-RS assessment, including saccadic intrusions during smooth pursuit, overshooting of saccades to a target, mild speech disturbance (slurring), slight postural tremor across both hands, movement slowing of both hands that was more pronounced for the left hand, a tremulous left-hand drawing, bradykinesia, and difficulties balancing during tandem walking that included three deviations. She reported mild difficulty getting up off the floor from lying on her back, walking on a slippery surface outdoors, walking in a dark room without falling, and getting dressed (pulling a shirt over her head). She exhibited some loss of sensation in her lower extremities, including a decreased vibration sense that was more severe on the left side.

In the MRI, hyperintensities were seen below the corpus callosum in the genu but not in the splenium. Several additional white matter hyperintensities were seen surrounding the lateral ventricles.

### 3.10. Case 10

Case 10 is a 66-year-old female premutation carrier with 62 CGG repeats. She did not report current concerns about FXTAS, though she noted during the medical history that she became concerned after falling multiple times in the span of 3–5 weeks in the last year. She has three biological children, including one biological daughter, who is a known premutation carrier and has no children. Case 10’s other daughter is a premutation carrier who has a son with FXS and a daughter with autism. The patient also has two biological sisters with the premutation (one also has been diagnosed with FXPOI) and one brother with the premutation. She has three nieces who are premutation carriers. She reports that neither of her parents was tested for the premutation, and she knows of no dementia or cognitive decline in her extended family. The only history of motor disease that she reports for her extended family is a maternal aunt who was diagnosed with Parkinson’s Disease.

During the clinical exam, Case 10 reported attention problems and that she is sometimes uncomfortable making eye contact during social interactions. She endorsed mild anxiety specific to social settings. She reports drinking 1–2 beers per day. She has had Raynaud’s syndrome since she was a teenager. She tested positive for scleroderma at age 52 years but then was retested, and this latter test was negative. She has also had a positive ANA test, but a retest was negative. She reports that sometimes she has grasping problems, during which her hand feels weak. Arthritis in the thumb was noted during the exam, which may be contributing to a weakened grip. Case 10 has fallen twice in the past year but not previously; one of these falls occurred when tripping on a gravel road during exercise. The patient reported mild difficulties walking on slippery surfaces outdoors, opening jars, and completing fine motor activities of daily living (e.g., picking up small objects). She reports memory problems (e.g., remembering names) starting around age 63 but indicates that she is not particularly concerned about memory changes. A systolic heart murmur during the medical exam was identified, and the patient reports she has known about this since childhood. During the FXTAS-RS, the patient showed dysmetria, Parkinsonian features including masked facies and bradykinesia, and brisk deep tendon reflexes. No other cognitive or memory issues were noted during standardized testing or the clinical exam. She did not demonstrate a tremor or ataxia, and tandem walking was normal.

In the MRI, white matter disease was observed in the splenium of the corpus callosum. General cerebral atrophy was evident.

See Table 2, Table 3, Table 4 and Table 5 for summarized information:

## 4. Discussion

Here, we report cases of aging women with premutation who are experiencing mild neurological symptoms that do not meet diagnostic criteria for FXTAS, and yet they have white matter disease observed in the MRI. The presence of white matter disease typically in the splenium of the corpus callosum is what is seen in the majority of female FXTAS cases [24,26], and therefore, this suggests that the pathophysiology of FXTAS in the brain has already begun. The FXTAS criteria were developed with male FXTAS patients (Table 1), although the splenium findings were added in 2014 [25] since this finding is seen in both males and females, and females do not usually demonstrate the middle cerebellar peduncle (MCP) sign, which is seen in the majority of males. We believe that the females reported here are already on the spectrum of FXTAS, but this overall diagnosis has a clinical meaning of more severe disease than what these women are presenting. Some of these women do not have tremors or ataxia; some have a subclinical presentation of tremors or balance problems or only intermittent symptoms that are mild by history. Therefore, they do not meet the full FXTAS criteria. For that reason, we propose the diagnosis of preFXTAS for these patients because they are at high risk of developing full FXTAS in time, and clinicians should consider treatment that may stall the onset of FXTAS in these patients. Such prophylactic treatments may include discontinuing alcohol, doing daily exercise, using antioxidants that are supplements to their diet, avoiding other toxins in their environment, and treating psychiatric problems [32,33] (see Table 4).

We have reported in the past males who showed the MCP sign, which is a major criterion for FXTAS [34], but these five males did not have tremors or ataxia, so they could not have been diagnosed with FXTAS, similar to the females reported here. However, one of the males reported [34] returned to our clinic with full FXTAS within 2 years, and others have developed more typical symptoms over time. A diagnosis of preFXTAS would have been appropriate for these males when they first presented, and similar findings were seen in a patient who presented with psychiatric problems and the MCP sign [35].

The presence of white matter disease (WMD) in the corpus callosum is sensitive to FXTAS but not specific to FXTAS because Hall et al. (2017) have demonstrated that it can be seen in other neurological disorders, including Parkinson’s disease, multiple system atrophy (MSA), etc., but it is more common in FXTAS than other neurological disorders [26]. The white matter disease in the corpus callosum (CC) was originally considered a major criterion when first added to the diagnostic criteria [25], but later, it was relegated to the minor radiological criteria by others [1,36].

Perhaps it is time to revisit the diagnostic criteria for FXTAS in females because they experience more significant problems with psychiatric symptoms at the beginning of FXTAS, which can cause excessive stress and may help to precipitate FXTAS in these women [1,5]. Loesch et al. have also emphasized that mild neurocognitive deficits can occur in aging females with the premutation before they experience tremor or ataxia [5]. In a recent evaluation of the progression of FXTAS symptoms comparing males and females, there was a significant difference in women vs. males with the premutation in that psychiatric problems, and even cognitive deficits can occur earlier in women compared to men with FXTAS [2,37].

In our study, we described several aging female carriers with neuropsychiatric problems and neurological abnormalities observed from the physical examination and brain MRI, which might be suggestive of preFXTAS. Although they have had normal or mildly abnormal neurological examinations, most of them were revealed to have white matter disease in the splenium in the MRI, while the classic middle cerebellar peduncle (MCP) sign, which is highly specific to males, was absent in these females [24,26]. The intention tremor and cerebellar gait ataxia, which are the major criteria for FXTAS, are also absent, or they are subclinical findings for these patients. These observations indicate that current diagnostic criteria may not fully capture the spectrum of FXTAS in females, and further review is required to reconsider new or modified criteria for FXTAS in females.

It is known that females with PM tend to have earlier psychiatric symptoms and more prevalent psychiatric signs, such as anxiety, mood disorders, and depression, compared to males with the premutation. [22,38]. Seritan et al. (2013) have found that the psychiatric problems in carriers start much earlier than the neurological problems associated with FXTAS [39]. In our study, 6 out of 10 presented with psychiatric diagnoses, and 3/10 of them with medications for these problems (most of them SSRIs), while motor (such as tremor or ataxia) and cognitive problems were absent or subclinical in them, except for memory problems. The most common problem observed in our case series was anxiety, which typically responds well to an SSRI and counseling. Active monitoring of anxiety, mood disorders, ADHD, and substance abuse should be undertaken in females with the premutation. All patients in our study presented with a normal IQ assessment at the time of their evaluation, but many had memory problems, and executive dysfunction deficits were often documented in the BDS2 studies (Table 4).

Autoimmune phenomena in female FXTAS patients are commonly observed but are still relatively under-recognized. Their presence can complicate the management of FXTAS. Previous studies have observed that FXTAS patients have an increased risk of autoimmune syndromes such as Rheumatoid Arthritis, Hashimoto’s Thyroiditis, Systemic Lupus Erythematosus (SLE), or fibromyalgia [1,40,41,42]. Coffey et al. [42] and Winarni et al. [43] showed autoimmune thyroid disorder, and fibromyalgia were found to be significantly increased in premutation women, particularly in those with FXTAS compared to age-matched controls [43,44]. The exact mechanisms behind this increased risk are not completely understood, but it is thought that the underlying genetic factors related to the premutation might play a role in both the neurodegenerative and autoimmune aspects. In the fragile X premutation, the *FMR1* gene has an expanded number of CGG repeats (55–200), which can lead to the sequestration of proteins important to mitochondrial and neuronal functions and the production of an abnormal protein, FMRpolyG, which is toxic to cells [1,45,46,47]. The toxicity of the premutation can dysregulate the immune system, which can lead to impaired immune tolerance (the immune system may lose its ability to differentiate between self and non-self), leading to autoimmune reactions and chronic inflammation. FXTAS has been associated with elevated levels of inflammatory markers in the blood [1], suggesting that inflammation may contribute to both neurological and autoimmune symptoms.

We believe that the recognition of preFXTAS females will allow early detection of the start of neuronal degeneration, and that will lead the clinician to recommend appropriate medication, lifestyle changes, and the avoidance of toxins in the early stages of the disease, with the aim to improve the outcome of neurocognitive, psychiatric, and other problems that are known to happen in advanced stages.

These findings suggest that there is a need for sex-specific modifications to the FXTAS diagnostic stages to accurately capture the appearance and progression of clinical findings in men and women. We propose referring to this symptomatic stage in women as preFXTAS. The difference between the initial symptoms observed in females and males in individuals with premutation has been described in the literature and was thoroughly analyzed in a recent study by Morrison D.E., et al. [37]. At this juncture, the establishment of sex-specific diagnostic criteria and early-stage involvement of females who may develop FXTAS is essential.

In this paper, we recommend the close monitoring of potential progression and neurodegenerative conditions among individuals showing features of preFXTAS. These individuals typically acquired some changes in white matter disease in the MRI examination of the brain, particularly in the splenium of the corpus callosum; however, there were limited neurological manifestations (with no or minimal tremors, ataxia) and nominal neuropsychiatric presentations. Due to this, they do not meet the criteria of FXTAS yet. This has also been reported in the past by Famula et al. [34] among males with *FMR1* premutation who do not exhibit clinical symptoms like tremors or ataxia but have already shown changes during the MRI brain studies, including the MCP sign Future research is required to explore this area of preFXTAS to allow for the early detection and better management of the individuals involved (Table 6). In addition, it is important to develop biomarkers for FXTAS to help in the diagnosis and to analyze the progression of FXTAS as new treatments are developed.

## Figures and Tables

**Table 1:** FXTAS diagnostic criteria in carriers of an *FMR1* premutation.

Level of Confidence	
Definite	Presence of 1 major radiological sign plus 1 major clinical symptom
Probable	Presence of either 1 major radiological sign plus 1 minor clinical symptom or has the 2 major clinical symptoms.
Possible	Presence of 1 minor radiological sign plus 1 major clinical symptom
**Symptom classes**	
**Radiologic:**	
Major	MRI white matter lesions in MCPs and/or brain stem
Minor	MRI white matter lesions in cerebral white matter
Minor	Moderate to severe generalized atrophy
Minor	MRI white matter lesions in the splenium of the corpus callosum
**Clinical:**	
Major	Intentional tremor
Major	Gait ataxia
Minor	Parkinsonism
Minor	Moderate to severe short-term memory deficiency
Minor	Executive function deficit
Minor	Neuropathy in lower extremities
**Neuropathology:**	
Major	FXTAS intranuclear eosinophilic inclusions that are ubiquitin-positive

From Tassone et al., 2023, Modified in Apartis et al. 2012, and Hall et al., 2014 [1,25,28].

**Table 2:** Molecular findings of the clinical cases: CGG repeats and Activation Ratio (AR).

Clinical Cases		Molecular Findings	
	Age	CGG Repeats	AR
1	71	30, 86	0.76
2	61	30, 69	0.22
3	72	29, 69	022
4	52	30, 114	0.90
5	75	20, 79	0.26
6	60	30, 114	0.42
7	70	31, 69	0.32
8	71	30, 102	0.9
9	78	31, 61	0.6
10	66	30, 62	0.6

**Table 3:** Clinical findings.

Clinical Cases		Neuropsychiatric Signs				Motor					Sleep Apnea	Autoimmune Disorder	BMI	Hypertension
	Age	Age at Onset	SCID (Psychiatric dx)	Medication	Memory Problems	Age at Onset	Balance and Gait Impairment	Subclinical Tremor	Clinical Intention Tremor	Clinical Rest Tremor				
1	71	69	Anxiety, depression	Fluoxetine	Yes	69	Yes	Intermittent once	No	No	Yes	No	28.1	No
2	61	59	Narcolepsy, sleep apnea, memory loss, anxiety	Dextroamphetamine—amphetamine, escitalopram	Yes	-	Normal	No	No	No	Yes	No	41	Yes
3	72	72	Depression, social phobia, specific phobia, PTSD	Sertraline	No		Yes	No	No	No	Yes	MCTD	23.2	No
4	52	Lifelong	Social phobia, specific phobia, generalized anxiety; obsessive-compulsive	None	Yes	49	Normal	Intermittent intention tremor	Yes	No	Yes	No	29	No
5	75	Not reported		None	Yes	73	Yes	Intermittent intention tremor	Yes (mild)	No	No	Yes (Raynaud)	24.0	No
6	60	NA	None	None	No	Lifelong	Yes	No	No	No	No	No	34.3	No
7	70	Not reported	Obsessive-compulsive; hostility, phobic anxiety; psychoticism (hostility is primary)	None	Yes	69	Yes	No	No	No	Yes	No	29.8	Yes
8	71	NA	None reported	None	No	Not reported	Normal	Yes	No	No	No	Yes (Raynaud)	24	No
9	78	Lifelong, formally dx at 76	OCD	None	No	Not reported	Yes	No	No	No	No	No	26.2	Yes
10	66	NA	Non reported	None	No	NA	Normal	No	No	No	No	Yes (Raynaud and scleroderma)	22.8	No

NA or non-reported; MCTD: mixed connective tissue disease (Sjogren, severe Raynaud’s, Rheumatoid arthritis); PTSD: post-traumatic stress disorder.

**Table 4:** Neurocognitive assessment.

Clinical Cases	WAIS					MoCA	MMSE	BDS2
	FSIQ	VCI	PRI	WMI	PSI			
1	126	127	113	117	127	25/30	30/30	20/27
2	*					21/30		23/27
3	98	103	81	111	105	30/30	**	23/27
4	105	98	105	95	121	22/30	25/39	15/27
5	130	118	125	122	129	28/30	27/30	24/27
6	114	99	129			NA	NA	24/27
7	99	90	107			NA	NA	22/27
8	116	128	116			NA	NA	23/27
9	111	113	106			NA	NA	25/27
10	116	123	105			NA	NA	21/27

* Case 2: She has Stanford–Binet: FSIQ 91, NVIQ 93, VIQ 90, ABIQ 94, FR 91, KN 94, QR 83, VS 100, WM 94; ** Not tested. NA is not available.

**Table 5:** MRI findings.

Clinical Cases	MRI										
	Cerebral Atrophy	Cerebellar Atrophy	Cerebral WMH	Cerebellar WMH	MCP Sign	PONS WMH	Sub-Insular WMH	Periventricular WMH	SPLENIUM CC WMH	GENU CC-WMH	CC Abnormal
1	Mild	No	No	No	No	No	Mild	No	Mild	No	Thin
2	Mild	Not mentioned	Yes	No	No	No	-	Yes	Minimum		No
3	Mild	No	No	No	No	Mild	Mild	Mild	Mild	No	No
4	Mild	No	Mild	No	No	No	No	No	Mild	No	No
5	No	No	Yes	Yes	No	No	No	Yes	Yes	No	No
6	No	No	Mild	Mild	No	No	Yes	Yes	Yes	Yes	
7	No	No	Yes	No	No	No	Yes	Yes	Yes	Yes	Thin
8	Yes	No	Yes	No	No	Mild	Mild	Mild	Mild	Mild	No
9	No	No	Mild	No	No	No	No	Yes	No	Yes	No
10	Yes	No	Yes	No	No	No	Yes	Yes	Yes	Yes	No

WMH: White Matter Hyperintensity; MCP: middle cerebellar peduncle; CC: Corpus callosum.

**Table 6:** Recommendations for females with the premutation and preFXTAS.

	Recommendations		
Hypertension and cardiovascular disorders	Treatment of hypertension; Follow up and cardiological assessment if murmur is founded	Physical exercise and Healthy balanced diet	
Obesity and overweight [48]	Physical exercise and Healthy balanced diet	Metformin [48]	Semaglutide [49]
Movement disorders and/or balance problems	Tremor: propranolol, primidone, topiramate, gabapentin; Ataxia: riluzole, Sinemet, Varenicline, Buspirone [33]	OT/PT. Help home support for daily life skills	
Psychiatric disorders	Active monitoring on anxiety, mood disorders, depression, ADHD, and substance abuse [22,50]	Psychotherapy; Physical exercise, Mindfulness meditation [33,51]	Selective serotonin reuptake inhibitors (SSRIs) or serotonin and norepinephrine reuptake inhibitors (SNRIs); buspirone; mirtazapine or CBD for insomnia; if needed, quetiapine (antipsychotic with less risk of extrapyramidal side effects) [52]
Cognitive and memory problems	Memantine is the only medication that has been studied in a controlled trial in FXTAS [33,53,54]	Donepezil, rivastigmine or galantamine (used for mild cognitive impairment or dementia) [33]	
ADHD	Counseling, Cognitive behavioral therapy (CBT)	Meds: Stimulants (methylphenidate or dexamphetamine) / non stimulants (atomoxetine, clonidine, guanfacine, modafinil)	
Hearing problem	Audiometry Follow up	Hearing aids often needed	
FXPOI, POI and POF [33]	Hormone replacement therapy (HRT)	Psychotherapy as needed; Emotional well-being and emotional support	Bone densitometry
	Vit. D and calcium supplements	Gynecological follow-up and family planning	Treatment for osteoporosis or osteopenia
OSAS or sleep apnea	Sleep study	CPAP	Weight loss
Chronic pain, fibromyalgia and chronic fatigue	Neuropathic pain: Gabapentin, pregabalin, topical cannabidiol (CBD) or Lidocaine patches [33]	Avoidance of opioids. Often helpful are SNRIs such as duloxetine or venlafaxine	Psychotherapy as needed
Osteoporosis [33]	Bone mineral density follow-up	Weight-bearing physical activity, healthy balanced diet	Calcium intake: 1000 mg/day 19–50 years old Estrogens; meds for osteoporosis
Sleep disorders	Exclude OSAS or sleep apnea	Exclude psychiatric disorders (es. ADHD and anxiety) [11]	Take into consideration: Mirtazapine 7.5 mg before sleep time CBD
Lifestyle	Vitamin supplements: Vit. D, Vit. B12, Folic acid, Vit. B6 Trolox (Vit. E analogue)	Physical exercise on a daily basis can be guided by PT, developed according to the needs and capacities of every individual [33]	Healthy balanced diet Eliminate alcohol or other toxins Eliminate opioids Eliminate smoking
	Antioxidants: green tea (epigallocatechin-3-gallate (EGCG), fresh berries, cabbage, omega 3s, curcumin, Vit. E, Vit. B9, melatonin, coenzyme Q10, ginseng, anthocyanins, N-acetyl-L cysteine (NAC), metformin, CBD [33,48]	Mindfulness based stress reduction (MSBR): mindfulness meditation, yoga and techniques for relaxation [33]	
Laboratory test to perform	Rheumatologist consult: ANA, C reactive protein (CRP), Ferritin	Thyroid function studies and thyroid replacement if low	
To avoid	Isoflurane in general anesthesia (If needed the safest option is propofol IV) or regional anesthesia [33]	Surgery	Drugs (marijuana, cocaine, methamphetamines, methadone)
	Alcohol	Smoking	Opioids

## Data Availability

Data can be obtained from the senior author R.J.H. upon request to rjhagerman@ucdavis.edu.
